# A meta-analysis to evaluate the cellular processes regulated by the interactome of endogenous and over-expressed estrogen receptor alpha

**DOI:** 10.18632/oncoscience.138

**Published:** 2015-03-07

**Authors:** Joana Simões, Francisco M. Amado, Rui Vitorino, Luisa A. Helguero

**Affiliations:** ^1^ Mass Spectrometry Centre, QOPNA Research Unit, Department of Chemistry, Universidade de Aveiro, Campus Universitário de Santiago, Aveiro, Portugal; ^2^ School of Healh Sciences, Universidade de Aveiro, Portugal; ^3^ Institute for Research in Biomedicine - iBiMED, Health Sciences Program, Universidade de Aveiro, Portugal

**Keywords:** estrogen receptor alpha, interactome, meta-analysis, breast cancer, mass spectrometry

## Abstract

The nature of the proteins complexes that regulate ERα subcellular localization and activity is still an open question in breast cancer biology. Identification of such complexes will help understand development of endocrine resistance in ER+ breast cancer. Mass spectrometry (MS) has allowed comprehensive analysis of the ERα interactome. We have compared six published works analyzing the ERα interactome of MCF-7 and HeLa cells in order to identify a shared or different pathway-related fingerprint.

Overall, 806 ERα interacting proteins were identified. The cellular processes were differentially represented according to the ERα purification methodology, indicating that the methodologies used are complementary. While in MCF-7 cells, the interactome of endogenous and over-expressed ERα essentially represents the same biological processes and cellular components, the proteins identified were not over-lapping; thus, suggesting that the biological response may differ as the regulatory/participating proteins in these complexes are different. Interestingly, biological processes uniquely associated to ERα over-expressed in HeLa cell line included L-serine biosynthetic process, cellular amino acid biosynthetic process and cell redox homeostasis.

In summary, all the approaches analyzed in this meta-analysis are valid and complementary; in particular, for those cases where the processes occur at low frequency with normal ERα levels, and can be identified when the receptor is over-expressed. However special effort should be put into validating these findings in cells expressing physiological ERα levels.

## INTRODUCTION

Sustained exposure to estradiol (E2) is associated with promotion and growth of breast cancer [1]. E2 action is mediated by estrogen receptors alpha and beta (ERα and ERβ, respectively). These proteins belong to the nuclear hormone receptor family of transcription factors, and are structurally divided into six functional domains A-F [2, 3]. The classical gene transactivation by ER, involves ER binding to its ligands (*e.g.* E2), change in ER conformation, dimerization and recruitment of co-regulator complexes at estrogen response elements (EREs), leading to transcriptional enhancement or repression of target genes. Nevertheless, ERs can also regulate gene expression through ERE-independent genomic action, without direct DNA binding, by modulating the function of other transcription factors through protein-protein interaction. The non-genomic ER actions are rapid and are frequently associated with the activation of various protein-kinase cascades in response to E2 [4, 5]. These non-genomic actions are possibly triggered through ERs located at the plasma membrane or, alternatively, associated to protein complexes located in the plasma membrane [6-8]. The genomic and non-genomic signal transduction pathways of activated ERs may connect with each other and involve multiple molecular complexes that modulate and/or mediate ER activity [9].

ERα is the main mediator of E2-induced proliferation and survival of breast cancer cells. Approximately 70% of breast cancers are ERα+ (ER+), and this receptor remains the primary target for endocrine therapies that aim to block ERα genomic and non-genomic actions with antagonists (tamoxifen, raloxyfen, ICI 182 780), or inhibiting E2 synthesis with aromatase inhibitors [10, 11]. Yet, about 50% ER+ breast cancers acquire resistance to these type of therapy through different molecular mechanisms that target ERα [11-13]. It is widely accepted that acquired endocrine resistance is associated to sustained growth factor receptor signaling leading to ER ligand-independent activation [10]. However, only 10-15% endocrine resistant breast cancers show this type of alteration [14, 15] which suggests that other mechanisms are at play when cancers acquire resistance [16].

The nature of the protein complexes acting in concert with ERs to control its functions in the cell, in particular upon ligand-independent or non-genomic ER activation is still an open question in breast cancer biology [17, 18]. Such information is of great interest because these proteins model the mechanisms of action of ERs involved not only in the promotion and progression of breast cancer, but also in the development of endocrine resistance. Further, they also constitute potential prognosis and follow-up markers. While in the past many laboratories have contributed to identify ER interacting proteins using a variety of experimental approaches and cell lines (a detailed list can be accessed at IntAct database: http://www.ebi.ac.uk/intact/query/estrogen%20receptor%20alpha?conversationContext=1), the recent advance of high resolution mass spectrometry (MS) has allowed for a more comprehensive analysis of breast cancer proteomes and interactomes [19-21] including the ERα interactome [17, 22-26]. Still, the cell type and context as well as the methodology used could lead to identification of different types of proteins /complexes, thereby hindering result extrapolation into generalized observations.

This study aimed to compare work carried out by others in which the ERα interactome was identified using two different cell lines: MCF-7 (ER+ breast cancer) and HeLa (ER-, cervical carcinoma) in order to identify a pathway-related fingerprint common to both cells lines or individually representing each of them. The MCF-7 cell line is an ideal model to study hormone response, whereas HeLa may not mimic the real ER signaling [27, 28]. This meta-analysis took into consideration the purification method, MS approach and whether ERα expression was endogenous or over-expressed.

## RESULTS AND DISCUSSION

A PubMed literature search identified six papers describing the ERα interactome obtained using different experimental approaches; of these, four used the ERα positive MCF-7 cell line [endogenous ERα expression (2 papers) or over-expressed ERα (2 papers)], one used the ERα negative HeLa cell line (over-expressed ERα) and one used both cell lines (over-expressed ERα). The experimental conditions for each paper analyzed are described in Table 1 and a list of the proteins identified can be found in Supplementary Table S1.

**Table 1:** Papers which identified ERα interacting proteins using MS analysis

#	Cell line	ERα	Treatment	ER Isolation	MS methodology	Ref
1	MCF-7	endogenous	5% DCC, 48hr	100nM E2, 37°C, 45', followed by ERE-Sepharose	2DE, MALDI, TOF/TOF	Nalvarte, et al
2	MCF-7	overexpressed	5% DCC, 5 days	10–8 M E2 added to protein extract 2hr prior ER isolation TAP-ERα	2DE (DIGE), nanoLC, ESI, QSTAR Elite hybrid Q/TOF-MS	Ambrosino, et al
3	HeLa	overexpressed	10% FBS	10μM E2, 10-20' followed by EREbinding and factionation in agarose gel	μLC, ESI, MS/MS	Schultz-Norton, et al
4	MCF-7	overexpressed	5% DCC, 5d	10 nM E2, 2hr followed by TAP-ERα	nanoLC, ESI, QSTAR Elite hybrid Q/TOF-MS	Tarallo, et al
5	MCF-7	endogenous	5% DCC+ E2, 3 days	Crosslinking folowed by Immunoprecipitation	nanoLC, LTQ Velos-Orbitrap MS	Mohammed, et al
6	MCF-7 HeLa	overexpressed	5% DCC + 10nM E2, 72hr	ERE-magnetic beads	1DE, nanoLC-MS/MS	Foulds, et al

Overall, the isolation methods used to purify ERα were variable with three using ERE-repetition oligonucleotides as bait for ERα pull down, two using TAP-ERα and one using ERα immunoprecipitation (Table 1). In addition, the cell treatment also varied between experiments with two papers treating cells with 10 nM E2 prior protein extraction and the remaining four adding E2 to the protein extract during the incubation reaction (Table 1). Given the difference in the number of proteins identified between these two conditions, we could not reliably compare these two types of treatments and for our purposes considered all identifications as in “E2-bound ERα”. In all experiments using MCF-7 cells, the growth medium was 5% DCC which allowed for better comparison between the results generated by the different labs. On the other hand, results from HeLa cell line were obtained with cells grown in 10% FBS, which may contribute to additional variability between the results. In all these studies ERα interacting proteins were identified using different MS-based proteomic analysis (Table 1), gel-free (paper 2, 3, 4, 5 and 6), gel-based (paper 1) and coupling both approaches (paper 2). Gel-based techniques rely in the protein separation using gel electrophoresis including one dimensional electrophoresis (1DE) and two-dimensional gel electrophoresis (2DE) [33, 34]. The 2DE has also its limitations like requirement of huge amount of sample, inability to detect low abundance proteins, to resolve highly acidic/basic proteins and proteins with extreme size and/or hydrophobicity, which is the case of membrane proteins [35, 36]. Additionally, technical bias induced by protein migration during the focusing step, or by gel staining, might difficult spot detection and their boundaries, making 2DE gel analysis a hard task which requires several replicates per sample [37]. Gel-free approach has been extensively developed and used in proteomic analysis because it allows overcoming some of the limitations of 2DE. This type of proteomic approach requires multiple liquid chromatography (LC) systems, essential to perform sample fraction of complex proteomes. Regarding disadvantages of MS, the low abundant proteins often will be lost because the mass spectrometer can only sequence a limited number of peptides in a given time window and these will normally be the most intense peaks, whereas low abundant peaks will not be sequenced although it is possible to partially overcome this problem [34].

Thus, in light of the high divergence between the experimental approaches used, we addressed the following questions with the aim to identify the processes, pathways and cellular components most represented: 1) What type of protein enrichment is associated to the isolation / MS approach?; 2) Is the ERα interactome different if ERα is endogenous or over-expressed in MCF-7 cells? and 3) Is there any difference between the ERα interactome in an ERα+ compared to an ERα-cellular background?

### Comparison between different ERα purification methodologies

**Figure 1:**
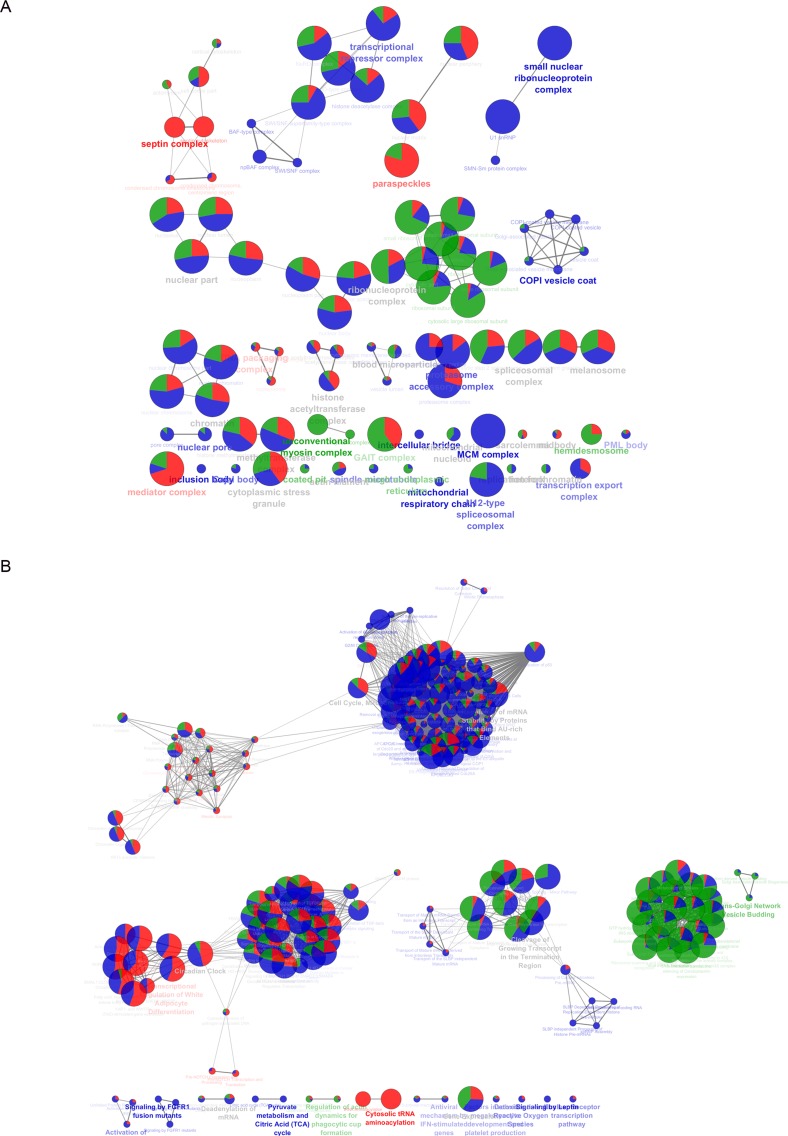
Comparison between MCF-7 cell ER interactome obtained with different purification methodologies: ERE-oligonucleotides (red), immunoprecipitation (blue) and TAP-ERα (green) The pie charts show the protein contribution from each methodology. A. Cellular component organization. B. Cellular pathways.

Comparison of ERα purification using ERE-oligonucleotides as bait [(paper 1 (ERE-Sepharose) and 6 (ERE-magnetic beads) in MCF-7 cells and paper 3 (ERE /Agarose) in HeLa cells; Table 1)] disclosed only one identification in common when comparing MCF-7 cells (NCOA2) and 15 shared identifications between MCF-7 using ERE-Sepharose and HeLa cells using ERE / Agarose (Supplementary Figure 1). This suggests that the different incubation procedures with E2, that is added during cell growth (paper 6) or added directly to the incubation reaction (papers 1 and 3; Table 1) possibly had an effect on the type of interacting protein isolated. Of a total of 96 (ERE), 129 (TAP) and 234 (IP) identifications in MCF-7 cells, 15 proteins were identified using ERE or TAP, 8 with ERE or IP, 4 with TAP and IP and none simultaneously by the three methodologies (Supplementary Figures 1 and 3). Further, when the cellular components represented by the three methodologies in MCF-7 cells were compared, Septin complex and paraspeckles were almost uniquely represented by ERE-purified ERα; GAIT complex and hemidesmosome by TAP-ERα, and mitochondrial respiratory chain, inclusion body, COPI vesicle coat, small nuclear ribonucleoprotein complex and MCM complex, were exclusively observed when ERα was immunoprecipitated (Figure 1A and Supplementary Table 2). Consequently, several pathways are differentially represented according to the ER purification methodology (Figure 1B and Supplementary Table 2).

This analysis clearly indicates that the methodologies used are complementary. Therefore, we proceeded to group the data in order to compare the interactomes of endogenous *vs* over-expressed ERα in MCF-7 cells and over-expressed ERα in MCF-7 *vs* HeLa cells.

### Interactome of endogenous and over-expressed ERα in MCF-7 cell line

**Figure 2:**
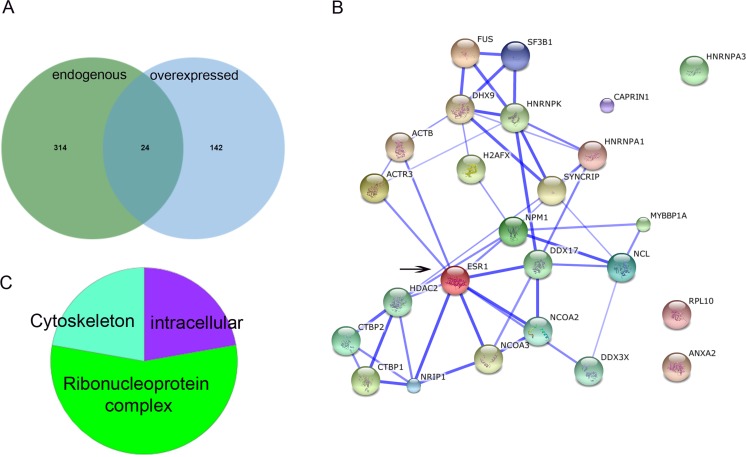
Interactome of MCF-7 cells expressing endogenous and over-expressed ERα A.Venn diagram representing overlapped and unique proteins interacting with endogenous or overexpressed ERα. B. Protein-protein interaction network extracted from STRING, depicting the shared interacting proteins between endogenous and overexpressed ERα (ESR1: arrow). The confidence view shows stronger associations represented by thicker lines and is based on co-occurrence, co-expression, experimental data and database. Medium confidence (Score=0.4). C. Cellular component distribution of shared interacting proteins between endogenous and overexpressed ERα according to http://pantherdb.org.

Three hundred and thirty-eight proteins were found associated to endogenous ERα and 166 proteins to over-expressed ERα; of these only 24 proteins were common to both conditions (Figure 2A and Supplementary Figure 3). The stronger interaction evidence according to STRING (Figure 2B) occurs directly with the co-repressors NRIP1 and HDAC2, and the co-activators NCOA2, NCOA3, DDX17 and DDX3X (SP1-dependent). Through interaction with the apoptosis suppressor NPM1, ERα is also associated to MYBBP1A and NCL1 which can either activate or repress transcription. Also, through NPM1, ERα may associate with SYNCRIP (heterogeneous nuclear ribonucleoprotein) and DHX9 (ATP-dependent helicase) both members of the CRD-mediated complex that promotes c-myc mRNA stability. The GO annotation in terms of cellular component (CC) using Panther (http://pantherdb.org) were cytoskeleton (22%), intracellular (22%) and ribonucleoprotein complex (56%). The biological processes most represented by these proteins where metabolic process (carbohydrate metabolic process, 7%; cellular aminoacid metabolic process, 7%, lipid metabolic process, 3.4%; nucleobase-containing compound metabolic process), 55%; and protein metabolic process (translation), 26%.

Proteins interacting only with endogenous ERα were localized at transcription export complex (TREX complex), proteasome accessory complex, small nuclear ribonucleoprotein complex and MCM complex (SupplementaryTable 3). In higher eukaryotes, the TREX complex recruitment is linked to mRNA splicing and/or capping [38, 39]. Even though, it is currently unclear whether the transport and processing of every mRNA occurs in the same way in every cell, this mechanism is conserved among species. Moreover, perturbations in the factors that are essential for mRNA nuclear export are linked to different diseases [40]. Therefore, the lack of representation by the over-expressed ERα interactome is surprising. The proteasome accessory complex caps one or both ends of the proteasome core complex and regulates entry into, or exit from, the proteasome; this may indicate differential turn-over of over-expressed ERα as compared to the endogenous protein. The MCM (mini chromosome maintenance) complex, has a role in both the initiation and the elongation phases of eukaryotic DNA replication, specifically the formation and elongation of the replication fork and are therefore essential for cell cycling. This could explain why MCF-7 cells transfected with ERα, fail to increase proliferation in response to E2 [41]. Yet, proliferation remains a major prognostic factor in ERα+ breast cancer [42] and these data shows a direct association between endogenous ERα and the MCM complex. In addition, a higher representation of cellular complexes associated to the nucleus and chromatin, including transcriptional repressor complex, histone deacetylase complex, U12-type spliceosomal complex and paraspeckles was also observed in the interactome of endogenous ERα. While the interacting proteins with over-expressed ERα were less represented in these complexes, except for a higher percentage representing the mediator complex (Supplementary Table 3). Mediator relays signals from transcription factors directly to the pol II enzyme, thereby regulating pol II activity and facilitating transcription factor-dependent regulation of gene expression. The lower % of these associated factors in the interactome of endogenous ERα, as well as the observation that MED15 and MED27 (interacting with endogenous ERα) are not found among the mediator complex proteins identified with over-expressed ERα is surprising, and suggests that gene regulation by ERα in this two settings differs.

The analysis of BPs showed that tRNA aminoacylation for protein translation was uniquely represented by the endogenous ERα interactome (Supplementary Table 3). Also, the interacting proteins with endogenous ERα showed more representation of protein folding, DNA metabolic process, chromosome organization, regulation of cell death and cellular response to DNA damage stimulus. On the other hand, the interacting proteins with over-expressed ERα were more representative of intracellular steroid hormone receptor signalling pathway, nuclear-transcribed mRNA catabolic process, nonsense-mediated decay (a quality control mechanism which eliminates mRNA transcripts with premature stop codons) and SRP-dependent co-translational protein targeting to membrane. The low representation of these two BPs by the interactome of endogenous ERα (23 and 20% vs 78 to 82% in over-expressed ERα) suggests that they possibly occur with lower frequency when ERα is expressed at physiological levels. Moreover, Reactome pathways including Cytosolic tRNA aminoacylation, RNA Polymerase I Promoter Clearance and Regulation of mRNA Stability by Proteins that Bind AU-rich Elements were significantly represented by proteins interacting with endogenous ERα; whereas Translation was significantly represented in the interactome of over-expressed ERα (Supplementary Table 3).

Regarding the lower representation of processes and complexes by over-expressed ERα, it can be argued that this is directly related to less number of proteins identified for this condition. However, even the proteins identified interacting with endogenous or over-expressed ERα for a defined process were, in most cases, not overlapping. Therefore, we conclude that in MCF-7 cells, the interactome of over-expressed ERα essentially represents the same BPs and CCs; however, the biological response may differ as the regulatory/participating proteins in these complexes are different. This may explain differential transactivation mechanisms between endogenous (ligand-dependent) and over-expressed ERα showing significant ligand-independent activity which may interact with mediator complexes in a ligand-independent manner, in a similar way as shown for over-expressed PPARs [43]. In addition, over-expression of ERs can lead to differential regulation of membrane proteins, such as cell adhesion proteins, through transcription-independent mechanisms [44, 45]. While, these effects possibly also occur at lower levels in the physiologic scenario [46, 47], over-expressed ERα results in higher interaction with SRP targeting to the membrane possibly making this effect more evident.

### Interactome of over-expressed ERα in MCF-7 and in HeLa cell lines

**Figure 3:**
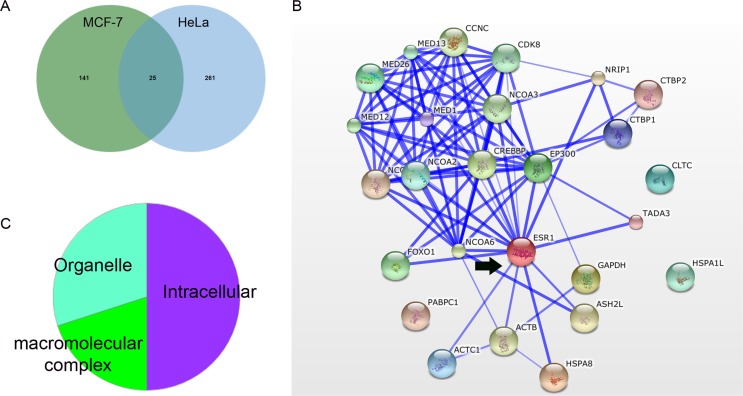
Interactome of MCF-7 and HeLa cells with over-expressed ERα A.Venn diagram representing overlapped and unique proteins interacting with overexpressed ERα in MCF-7 and HeLa cells. B. Protein-protein interaction network extracted from STRING, depicting the shared interacting proteins between MCF-7 and HeLa cells ERα (ESR1: arrow). The confidence view shows stronger associations represented by thicker lines and is based on co-occurrence, co-expression, experimental data and database. Medium confidence (Score=0.4). C. Cellular component distribution of shared interacting proteins between overexpressed ERα in MCF-7 and HeLa cells according to http://pantherdb.org.

One-hundred and sixty-six proteins were identified interacting with ERα in MCF-7 cells and 286 proteins interacting with ERα in HeLa cells. Only 25 proteins were shared by both cell lines (Figure 3A and Supplementary Figure 4). Among the shared interactors by MCF-7 and HeLa cells were components of the mediator complex (CCNC, CDK8, MED1, MED12, MED13, MED26), several co-activators (NCOA1, NCOA2, NCOA3, NCOA6, CREBBP, FOXO1, EP300, TADA3) and co-repressors (NRIP1, CTBP1, CTBP2) all which could directly interact with ERα (Figure 3B). These proteins were intracellular (50%), organelle (of which 66% are in the cytoplasm and 33% in the nucleus), and macromolecular complexes (20%, including protein and ribonucleoprotein complexes) (Figure 3C). The BPs over-represented according to Panther were cell process (cell communication, cell cycle, cell proliferation and cytokinesis), 17%; biosynthetic process (carbohydrate metabolic process, cellular amino acid metabolic process, nucleobase-containing compound metabolic process and protein metabolic process), 30%; and biological regulation (regulation of transcription from Pol II promoter), 17%.

The CC by ERα interacting proteins in MCF-7 cells uniquely represented unconventional myosin complex and hemidesmosome (Supplementary Table 4), suggesting a role of ERα in regulating cell movement and localization at the cell adhesion contacts. Experimental evidence suggesting such regulation may be independent of ER transcriptional activation has been reported [48, 49]. Catalytic step 2 spliceosome, mediator complex and ribonucleoprotein complex were also more represented by the interactome from MCF-7 cells. On the other hand, protein phosphatase type 2A complex (PP2A), proteasome complex, eukaryotic translation elongation factor 1 complex and COPI vesicle coat were uniquely over-represented by the interactome of HeLa cells. PP2A expression is upstream of ER expression and contributes to the ERα+ phenotype in breast cancer cell lines through stabilization of ER mRNA [50] and ER-non genomic activation of PP2A has also been shown in vascular smooth muscle cells (with endogenous ER) [51]. COPI coated vesicles are involved in retrograde transport to the endoplasmic reticulum from the Golgi network, again suggesting that ERα regulates membrane proteins.

Among BPs, the ones represented by similar amount of proteins in MCF-7 cells compared to HeLa cells were related to RNA regulation, including: intracellular receptor signalling pathway, nucleobase-containing compound catabolic process, mRNA metabolic process and posttranscriptional regulation of gene expression (Supplementary Table 4). Histone acetylation and protein folding were mostly represented by the HeLa interactome. Interestingly, BPs uniquely associated to ERα over-expressed in HeLa cell line included L-serine biosynthetic process, cellular amino acid biosynthetic process and cell redox homeostasis, clearly suggesting that in this ERα-background, many proteins involved in cell metabolism and energy homeostasis can interact with over-expressed ERα, with consequences regarding cell growth and proliferation. Protein deneddylation was also uniquely represented by the HeLa interactome. Deneddylation of proteins removes the Nedd8 moiety from cullins, thereby preventing cullins from organizing ubiquitin ligase (E3) complexes in order to target cellular proteins for proteasomal degradation [52]. As ERα is itself targeted for proteasomal degradation by ubiquitin E3 ligases [53], in HeLa cells over-expressing ERα, this interaction may favour ERα stability.

The interactome in HeLa cells was associated to pathways rarely observed in MCF-7 cells: Glycolysis, Prefoldin mediated transfer of substrate to CCT/TriC, Destabilization of mRNA by AUF1 (hnRNP D0), Cytosolic tRNA aminoacylation, Sulfur amino acid metabolism. Regarding the ERα interactome of MCF-7 cells, the association to transcription and mRNA splicing was more represented than in HeLa cells (Supplementary Table 4). Gene expression was equally represented in both cell lines; however, the proteins associated to these pathways were in most cases not over-lapping.

## CONCLUSION

In this work we addressed the question of whether endogenous and exogenous ERα interact with similar cellular proteins and regulate the same biological processes. This question is relevant, given that for analysis of interacting proteins and gene expression, over-expressed ERα is generally used. Indeed, with the over-expressed receptor, the biological effects observed are clearer than when analyzing endogenous ERα levels. Nevertheless, they may not reflect the physiological interactions occurring in ERα expressing cells. On the other hand, it can be argued that those interactions occur naturally, but at such low frequency that they are not detected during MS sequencing.

The different purification methodologies will enrich in specific ERα containing complexes and therefore highlight different pathways. Interestingly, the ERα interactome disclosed ERα possible presence in complexes across all cell organelles and subcellular localizations; thus, supporting the idea that ERα is not merely a transcription factor and that its activation leads to biological effects regulated transcriptionally and non-transcriptionally.

Therefore, while all the approaches analyzed in this meta-analysis are valid and complementary, special effort should be put into validating findings from cell lines with over-expressed ERα in cells expressing natural levels of the receptor. Such actions will no doubt advance our understanding of ER signalling and uncover novel potential targets to revert endocrine resistance.

## METHODS

In order to analyze the ERα interactome, database searching was carried out on PubMed, Google Scholar and Web of Knowledge using the keywords “ERα interactome”, “ERα interacting proteins”, “ERα associated proteins”, “ERα interactors” and “ERα mass spectrometry” for articles studying the interactome of ERα using MS in breast cancer cell lines or mammary epithelial cells. Six papers were identified which contained a full and comprehensive list of interacting proteins and which were used for this meta-analysis. For each identification, the accession number, protein name and gene name were extracted from the Uniprot (http://www.uniprot.org/) and Panther (http://www.pantherdb.org/) databases (Table 1 and Supplementary Table S1). In order to identify statistically over-represented Gene Ontology terms corresponding to Biological Process (BP), Cellular Component (CC) and pathways, we used the Cytoscape 3.1.0 (http://www.cytoscape.org/) [29] software with ClueGo v2.1.5 and CluePedia v1.1.5 plugins [30, 31]. In all analysis, filters were set to Go tree interval with 3 minimum and 8 maximum levels; Go term/Pathway selection with 3 minimum genes and 8% genes; medium network specificity (Kappa score = 0.4) and over-representation was considered significant if *p*<0.05. STRING database 9.1 (Search Tool for the Retrieval of Interacting Genes) [32] (http://string-db.org/) was also used to provide a grid of possible ERα containing protein complexes (Kappa score = 0.4).

## SUPPLEMENTARY MATERIALS FIGURES AND TABLE




