# Supplementary material for: A meta-analysis to evaluate the cellular processes regulated by the interactome of endogenous and over-expressed estrogen receptor alpha
Source: Oncoscience. 2015 Mar 7;2(5):487–96. doi: 10.18632/oncoscience.138 (PMC4468335; doi:10.18632/oncoscience.138)
Supplement: Supplementary file 1 [file oncoscience-02-0487-s001.pdf]

A meta-analysis to evaluate the cellular processes regulated by the interactome of endogenous and over-expressed estrogen receptor alpha

Supplementary Material

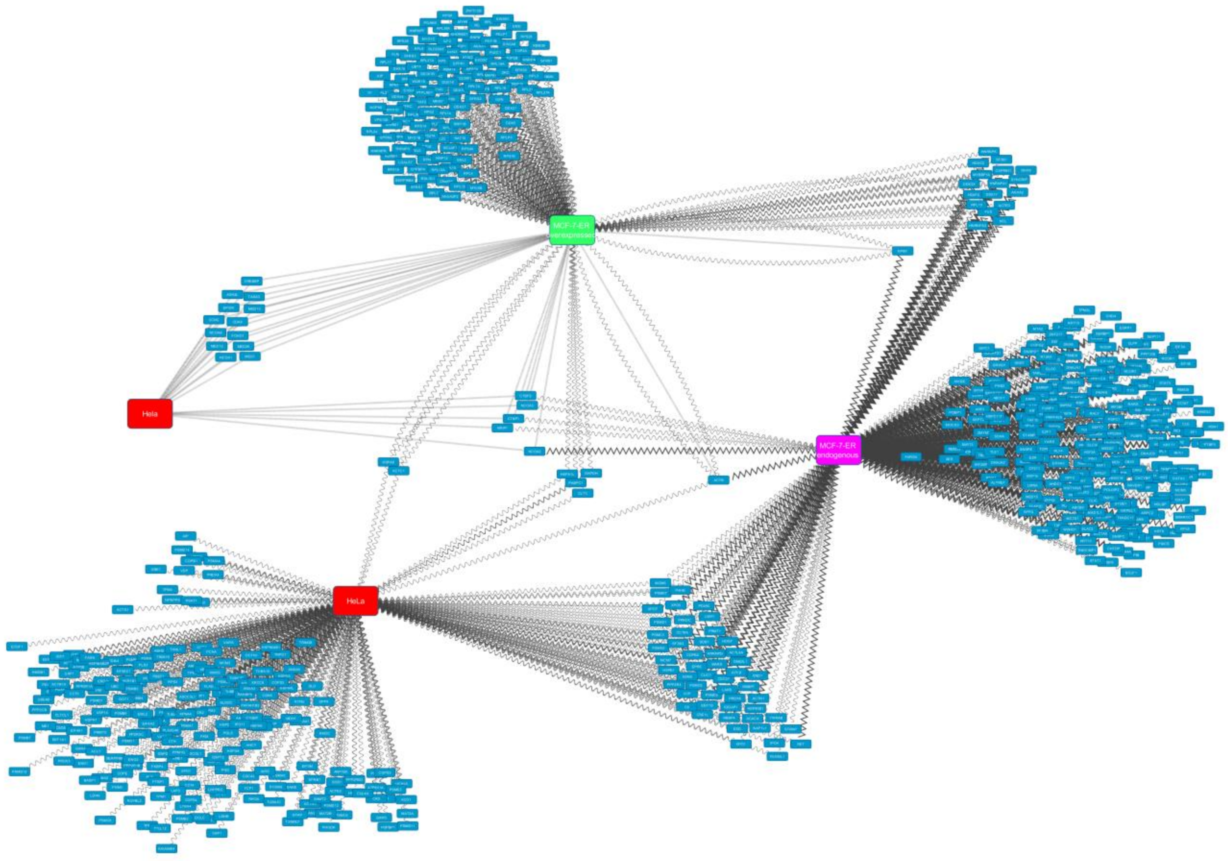

Supplementary Figure 1: A network visualizing the correlation between the ERα interacting proteins purified by three different methodologies: ERE-repetition oligonucleotides (ERE); TAP-ERα (TAP) or immunoprecipitation (IP) and identified using MS approaches. Backward slash (gel-free-based approach), zig-zag (gel-based approach), sine wave (gel-free approach). The network was constructed using the Cytoscape platform. $p<0.05$

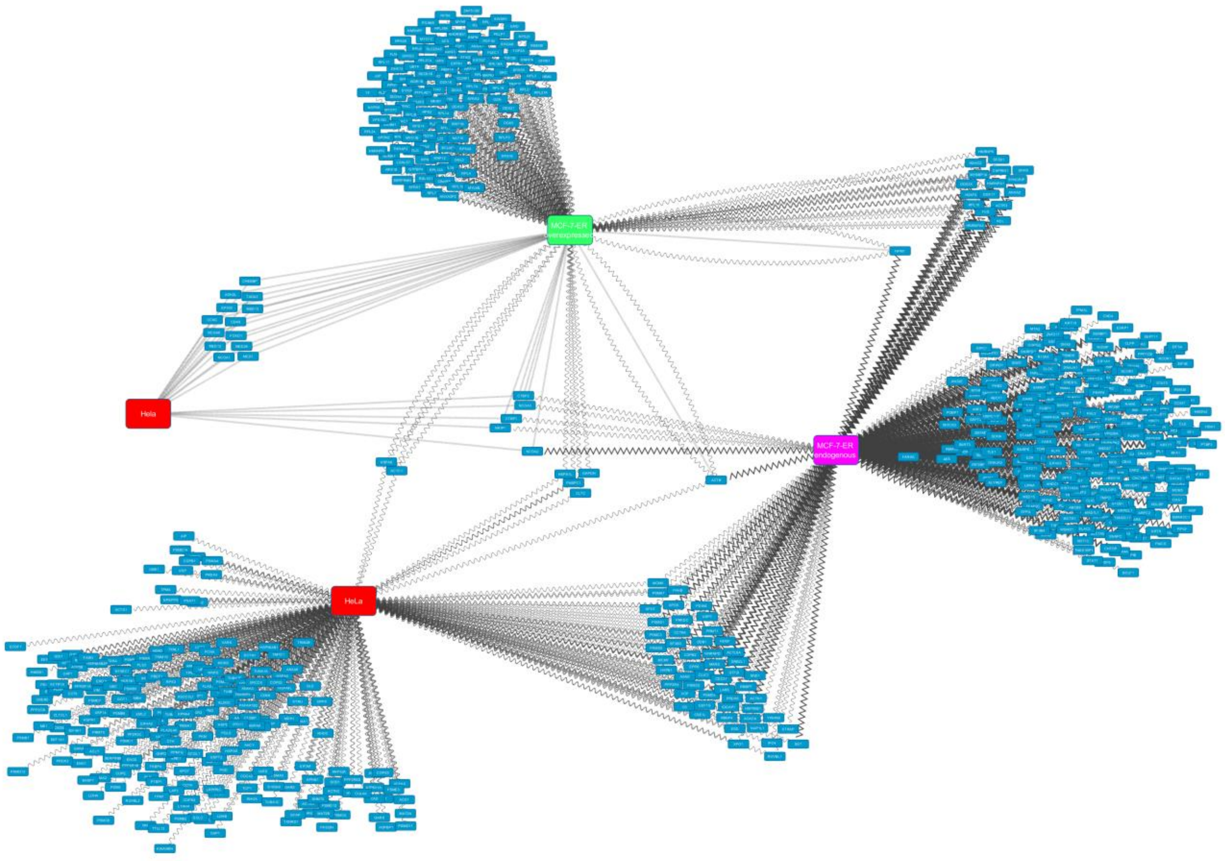

Supplementary Figure 2: A network visualizing the correlation between the ERα (over-expressed and/or endogenous) interacting proteins in MCF-7 and HeLa cell lines using MS approaches. Backward slash (gel-free-based approach), zig-zag (gel-based approach), sine wave (gel-free approach). The network was constructed using the Cytoscape platform. $p<0.05$

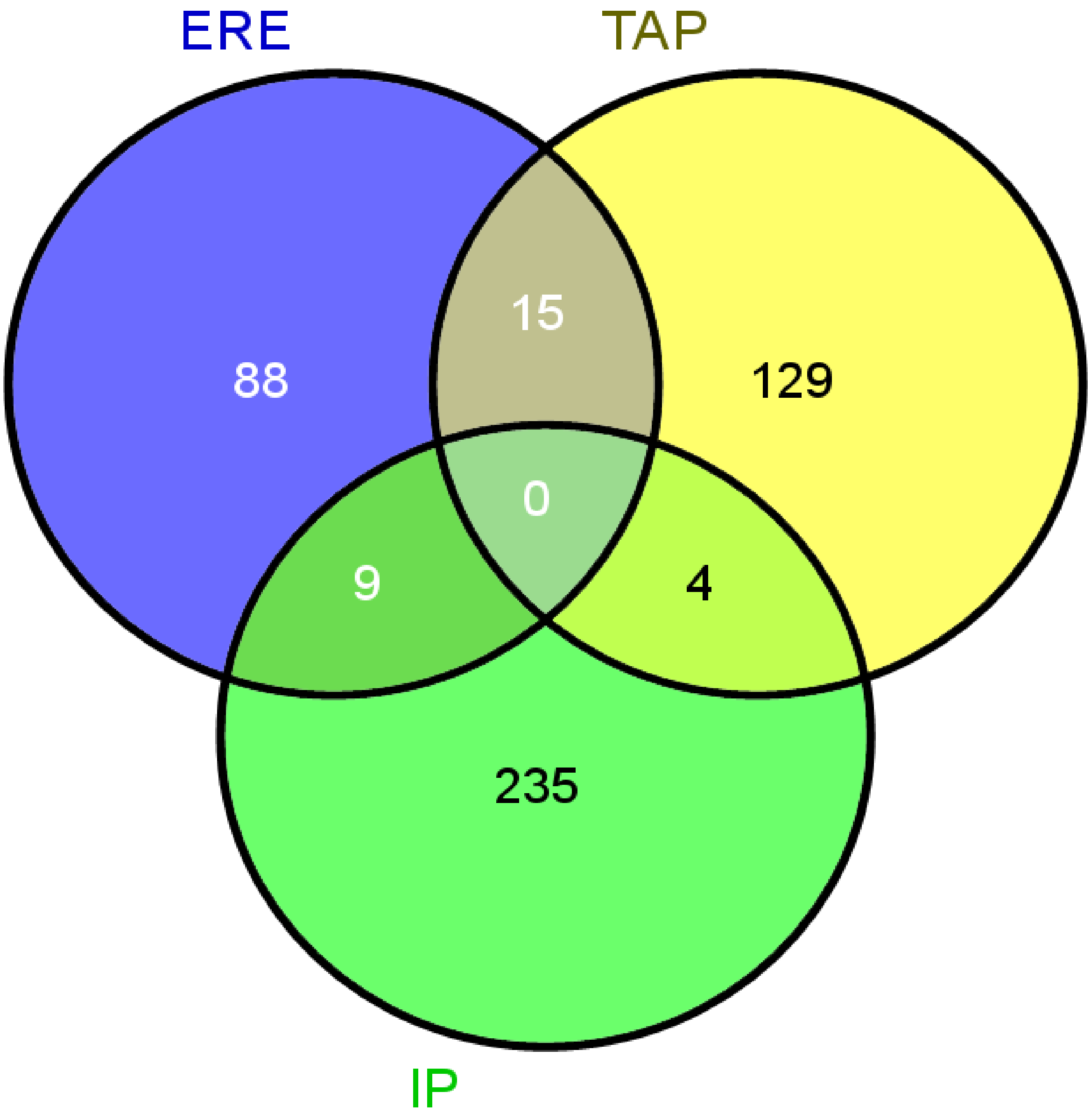

<u>Supplementary Figure 3:</u> Venn diagram showing the unique and shared identifications using three different methodologies to purify ERα in MCF-7 cells: ERE-repetition oligonucleotides (ERE); TAP-ERα (TAP) or immunoprecipitation (IP).
